# The Effect of Enteral Nutrition Support Rich in TGF-β in the Treatment of Inflammatory Bowel Disease in Childhood

**DOI:** 10.3390/medicina55100620

**Published:** 2019-09-22

**Authors:** Mehmet Agin, Aylin Yucel, Meltem Gumus, Hasan Ali Yuksekkaya, Gokhan Tumgor

**Affiliations:** 1 Department of Pediatric Gastroenterology, Hepatology and Nutrition, Cukurova University Medical Faculty, Saricam, 01380 Adana, Turkey; gtumgor74@yahoo.com; 2 Department of Pediatric Gastroenterology, Hepatology and Nutrition, Necmettin Erbakan University Medical Faculty, Meram, 42080 Konya, Turkey; ayucel82@hotmail.com (A.Y.); meltemdorum@gmail.com (M.G.); dryuksek@yahoo.com (H.A.Y.)

**Keywords:** pediatrics, growth retardation in IBD, Crohn disease, ulcerative colitis, modulen IBD

## Abstract

*Background and Objective:* Malnutrition is a major complication of inflammatory bowel disease (IBD). Our aim of the study was to examine the effects of Modulen IBD supplementation, which was administered to IBD patients without limiting their daily diet in addition to medical treatment, on the clinical, laboratory, anthropometric values, and disease activities of these patients. *Materials and Methods:* Seventy three children with IBD were evaluated retrospectively. The cases were classified as those who had Crohn disease receiving (CD-M; *n* = 16) or not receiving Modulen IBD (CD; *n* = 19) and those who had ulcerative colitis receiving (UC-M; *n* = 13) or not receiving Modulen IBD (UC; *n* = 25). Disease activities, laboratory values, remission rates, and anthropometric measurements of the groups were compared. In addition to IBD treatment, Modulen IBD in which half of the daily calorie requirement was provided was given for eight weeks. *Results:* In the third month of treatment, 14 (88%) patients were in remission in CD-M group and eight (42%) patients were in remission in CD group. The height and weight z scores, which were low at the time of diagnosis, improved in the first week in CD-M group. Inflammatory parameters (UC) were significantly lower in the UC-M group compared to the UC group in first and third months. In the third month, eight (62%) patients in the UC-M group and four (16%) in the UC group were remitted clinically and in terms of laboratory values. *Conclusions:* TGF-β-rich enteral nutrition support in children with IBD is an easy, effective, and reliable approach. It was shown that TGF-β-rich enteral nutritional supplementation enabled the disease to enter the remission earlier, and contributed to the early recovery of weight and height scores.

## 1. Introduction

Growth retardation and malnutrition is a major complication seen in children with inflammatory bowel disease (IBD). Approximately 90% of children have weight loss at the time of diagnosis [1]. Growth retardation is less common in patients with ulcerative colitis (UC) than in patients with Crohn’s disease (CD), but growth retardation is observed in both groups [2]. In the development of malnutrition in patients with IBD, various factors play a role such as malabsorption, increased intestinal loss, catabolic effects of systemic inflammation, and anorexia nervosa developed due to the chronic inflammation in bowel [3]. Nutritional support plays an important role in the prevention of complications such as malnutrition, osteoporosis, and micronutrient deficiency in the treatment of children with IBD. Nutritional support therapy has been reported to have a direct effect on the intestinal mucosa by suppressing the production of cytokines and inflammation [4,5].

TGF-β (transforming growth factor) is a multifunctional key regulatory peptide which is released from different cell types. The best-known effects of TGF-β are immunoglobulin, differentiation, and cell growth. Like many similar cytokines in intestinal tissue, TGF-β may also play a critical role in anti-inflammatory processes, prevention of autoimmunity, and tolerance mechanisms [6].

Modulen IBD^®^ (Nestle, Vevey, Switzerland) is a rich in TGF polymeric liquid formula. Nestle made the new product (CT3211) in a variety of flavors and marketed it as Modulen IBD in the summer of 2001. In 2001, Modulen IBD was also initiated to be used in the treatment of IBD [7].

In our clinical practice, it has been used in patients with IBD for approximately five years, in addition to the normal diet and received medical treatments, both at the time of diagnosis and relapses. Generally, enteral nutrition solutions, elemental (amino acids mixture), semi elemental (hydrolyzed proteins), and whole protein (polymeric) formulas are administered with oral or nasogastric tubes. The taste of polymeric formulas is better and they are cheaper. They are used successfully especially in children with CD [4].

The use of enteral feeding solutions decreases the need for corticosteroids and reduces the side effects of corticosteroids in patients who have IBD, especially in those with CD. The presence of nutritional deficiencies in children who have CD and UC affects nutritional balance, bone development, growth and sexual development, oxidative stress and defense mechanisms, tissue repair, immune system, and clinical recovery [8,9].

In addition to various anti-inflammatory drugs that are used in IBD treatment, it is considered that additional nutritional supplements, which are administered to such patients who are usually malnourished and who have lack of appetite are effective in their remission. There are very few studies in which Modulen IBD given additionally without restricting daily diets in children with CD is examined. Unlike the studies conducted on CD, we could not find any studies conducted on this subject in patients with UC. In the present study, our purpose was to examine the effects of Modulen IBD supplementation, which was administered to IBD patients without limiting their daily diet in addition to medical treatment, on the clinical, laboratory, anthropometric values, and disease activities of these patients.

## 2. Materials and Methods

The medical records of 85 patients with IBD (41 cases CD, 44 cases UC) followed-up between January 2012 and January 2016 at Pediatric Gastroenterology, Hepatology and Nutrition Departments of Cukurova University Faculty of Medicine (Adana, Turkey) and Necmettin Erbakan University Faculty of Medicine (Konya, Turkey) were reviewed retrospectively. Two patients with CD were excluded from the study because they could not tolerate Modulen IBD orally, two patients were excluded because they had incomplete data in their files, and two patients were excluded because they had a history of surgery. Two patients with UC were excluded from the study because they could not tolerate Modulen IBD orally, and four patients were excluded from the study because they had incomplete data in their files. The cases that had active disease were accepted for the study.

The patients who had missing upper and lower gastrointestinal endoscopies, missing histopathological, clinical, and demographic data, who could not tolerate Modulen IBD orally, who had undergone intestinal surgery, and who had previously received Modulen IBD treatment medically were excluded from the study.

The remaining 73 patients who had IBD were included in the study. In the group that was diagnosed with CD, 12 patients had ileum and colon involvement in the CD-M group, and four patients had isolated ileum involvement. In the CD group, 14 cases had ileum and colon involvement, and five cases had isolated ileum involvement. In the group that was diagnosed with UC, 11 patients had pancolitis in the UC-M group, and two patients had distal colitis. In the UC group that did not receive Modulen IBD, 21 patients had pancolitis, and four patients had distal colitis. Medical treatment was started in patients with CD as steroid 2 mg/kg/day (maximum 60 mg/day) and Mesalazine 50–150 mg/kg/day (maximum 3 g/day) was started for the cases that were diagnosed with UC. In addition, proper antibiotic treatment was given to the patients who had infection. Modulen IBD was initiated for patients who had malnutrition and more severe disease.

The cases were classified as CD receiving Modulen IBD (CD-M; *n* = 16) and those who did not receive Modulen IBD (CD; *n* = 19), and UC receiving Modulen IBD (UC-M; *n* = 13) and those who did not receive Modulen IBD (UC; *n* = 25). The treatment of the cases with ulcerative colitis and with CD is regulated according to ECCO/ESPGHAN recommendations [10,11]. Modulen IBD was administered for eight weeks in addition to their normal diet and medical treatments (in addition to steroid treatment in patients with Crohn disease and mesalazine treatment in patients with ulcerative colitis to form half of the calories required to be taken daily. Modulen IBD which is from the protein source casein, is a polymeric enteral nutrition solution rich in TGF β. Its protein content is 14%, carbohydrate content is 44%, and fat content is 42%. The carbohydrate source is sucrose and glucose polymers. It does not contain lactose. The fat content includes 55.6% milk fat, 13.9% corn oil, and 26.1% medium chain triglycerides. Its osmolality is 312 mosm/L and contains sufficient amounts of vitamins, trace elements, and minerals. Patients are advised to drink 50 g of Modulen IBD in approximately half an hour after it is diluted with 210 mL of fresh water. The preferred energy quantity of the Modulen IBD is to receive more than 210 calories per meal.

Colonoscopy was performed for all of the cases using Pentax EG-2730 K Colonoscopy Device (Pentax, Tokyo, Japan) after the routine bowel cleansing and the sedation administration with midazolam zero,1 mg/kg and profol 1 mg/kg/dose by an anesthesiologist after 8-h fasting.

For histopathological examination, multiple biopsies were taken from colon and ileum mucosa of all cases and placed in solutions containing 10% formol. All biopsies were examined by a pathologist specialized in gastroenterology. The diagnosis of CD and UC was made according to the clinical, histopathological, and endoscopic criteria determined by the European Society for Pediatric Gastroenterology Hepatology and Nutrition (ESPGHAN) [12].

Pediatric Crohn Disease Activity Index (PCDAI) was used for the disease activity of patients with CD; and Pediatric ulcerative colitis activity index (PUCAI) was used for the disease activity of the patients with UC [13,14].

Weight, height, hemogram, biochemistry, erythrocyte sedimentation rate (ESR), CRP, IgG, PCDAI, PUCAI, and remission rates were evaluated statistically by comparing among the groups at the time of diagnosis, in the first week, and in the first, third and sixth months. The absence of clinical symptoms in patients with both CD and UC and the decrease in PCDAI and PUCAI below 10 was accepted as the remission criterion [13,14]. The body weight and height were measured using standard anthropometric techniques [15].

Weight and height standard deviation scores were calculated using a software Package (LG ROW, Child Growth Foundation, London, United Kingdom) and were expressed as weight for age z score (WAz) and height for age z score (Haz), retrospectively [16].

### 2.1. Patient Informed Consent and Ethics Committee Approval

Verbal and written informed consents were obtained from all the subjects included in the study and from their parents. After the study was completed, the study result of each subject was reported to his/her own parents. Ethics committee approval for the study was given by Cukurova University Medical Faculty, Clinical Research Ethics Committee (Adana, Turkey, Approval number: 38/62, approved on 3 March 2017).

### 2.2. Statistical Method

Normality of numerical data tested by Shaphiro–wilk test; and Mann Whitney U test was used for non-normal data to compare two independent groups. Relationship between categorical variables was tested by the Chi-square test; and RR values and 95% confidence intervals were calculated. Frequency, percentage (%) and mean ± standard deviations were given as descriptive statistics. All analyses were performed by using SPSS for windows version 24 and a *p* value smaller than 0.05 was considered significant.

## 3. Results

A total of 73 children who had IBD were retrospectively evaluated and were classified as those with Crohn disease who received Modulen IBD (CD-M; *n* = 16) or who did not receive Modulen IBD (CD; *n* = 19) and those with UC who received Modulen IBD (UC-M; *n* = 13) or those who did not receive Modulen IBD (UC; *n* = 25).

### 3.1. In the Cases with CD

In the CD-M cases included in the study, four (25%) were female and 12 (75%) were male. In the CD group, nine (47%) were female and 10 (50%) were male. The mean age was 14.1 ± 3.7 (Range 10–17.8 years) years in CD-M group and it was 14 ± 3.5 (Range 10–17.7 years) in CD group (*p* = 0.17).

#### 3.1.1. Inflammatory Parameters

The hemoglobin and albumin values of the CD-M group at the time of diagnosis were lower than the CD group. The platelet counts were significantly higher (*p* < 0.05). Total protein, sedimentation, and CRP levels were high in both groups and no statistically significant difference was found between them. In the first month, albumin levels were normalized in the CD-M group and a statistically significant decrease was observed in ESR compared to the CD group. Inflammatory parameters normalized in the third month, and no statistical difference was found between the two groups (Table 1).

#### 3.1.2. Disease Activity

PCDAI was higher in the CD-M group at the time of diagnosis than in the CD group. PCDAI started to decrease more rapidly in the CD-M group, and it was significantly lower in the first and third months compared to the CD group (*p* < 0.05). In the sixth month, it was within normal limits in both groups (Table 2).

In the CD-M group, only one patient was in complete remission in the first month while no one was in remission in the CD group. In the third month, 14 (88%) patients were in remission in the CD-M group, whereas eight (42%) patients in the CD group were in complete remission, which was statistically significant (*p* < 0.05). All patients were in remission in the sixth month (Table 2).

In the CD-M Group, all the patients (16/16) improved in clinical terms in the first month; however, only five (5/19) patients had clinical improvement in the CD group. Clinical improvement was detected in the third month in all cases of both groups.

#### 3.1.3. Anthropometric Measurements

The average weight z score of the CD-M group at the time of diagnosis was −2.2 SDS (Standard Deviation Score) and had severe malnutrition. At the time of diagnosis, the weight-for-age z score in the CD-M group was significantly lower than in the CD group (*p* < 0.05). It the first week of follow-up, it began to improve rapidly. No difference was observed between the two groups in the first, third, and sixth months. No significant difference was found in weight-age z scores (Table 2).

### 3.2. In the Cases with UC

Five (38%) of the patients in the UC-M group were female and eight (62%) were male; and 10 (40%) were female and 15 (60%) were male in the UC group. The mean age was 12.9 ± 3.5 years (Range 9.4–16.5 years) in the UC-M group and 14.4 ± 3.4 (Range 11–17.9 years) in the UC group (*p* = 0.007).

#### 3.2.1. Inflammatory Parameters

At the time of diagnosis, thrombocyte levels were found to be higher in the UC-M group and albumin values were found higher in the UC group. No statistically significant difference was observed between the other parameters. ESR and CRP values were significantly lower in the UC-M group in the first and third months than in the UC group. No statistically significant difference was determined in the sixth month (Table 3).

#### 3.2.2. Disease Activity

There was no significant difference between the two groups at the time of diagnosis. PUCAI was found to be significantly lower in the UC-M group in the first week compared to the UC group. In the first and third months, PUCAI values were significantly lower in the UC-M group than in the UC group. In the sixth month, no statistically significant difference was observed between them. In the first week, none of the cases in the two groups were in remission, whereas in the UC-M group, three patients were in remission. In the third month, eight (62%) cases in the UC-M group and four (16%) in the UC group were in remission and a statistically significant difference was found. In the sixth month, all cases in the two groups were in remission (Table 4).

All the patients (13/13) improved clinically in the first month in the UC-M Group; however, only eight patients (8/25) improved clinically in the UC group. Clinical improvement was detected in all cases of both groups in the third month.

#### 3.2.3. Anthropometric Measurements

Significant improvement was observed in the UC-M group in the first and third months of weight-for-age and height z scores. Weight and height z scores were improved in all cases in the third and sixth months and no statistically significant difference was found (Table 4).

### 3.3. The Side Effects of Modulen

Right after starting Modulen IBD, three patients who had CD-M had mild abdominal pain and abdominal distention, and two patients who had UC-M had nausea. The minor complaints of these cases regressed in a short time. No serious side effects were seen in any patient.

## 4. Discussion

IBD constitutes a group of diseases which causes malnutrition. At the time of diagnosis of children with CD, weight loss is observed in 85% and the length of the disorder is seen in 46% [17]. Inflammation, malnutrition, and corticosteroid treatments are important etiologic factors for growth retardation in pediatric IBD. Concerns about the side effects of corticosteroids related to mucosal healing, bone mineral density, and growth are increasing [18,19,20]. This situation increases the importance of enteral nutrition; and therefore, a good and adequate nutritional support is needed in IBD. The main purpose of nutrition in children with IBD (especially the children with CD) is to provide the disease to be in remission and to perpetuate the growth and development by maintaining remission [21].

In experimental studies on animals, Modulen IBD support has been reported to be protective against gastrointestinal tract damage, acidosis, hypoalbuminemia, and weight loss [22]. The clinical response to Modulen IBD is associated with the down regulation of mucosal proinflammatory cytokine mRNA and mucosal healing in both colon and terminal ileum [22]. An important anti-inflammatory effect of TGF β is the promotion and generation of FOXP3 positive regulatory T cells in the intestinal compartment [23].

There is no consensus on Enteral Nutrition (EN) content and duration of use in pediatric centers where EN is used. No difference was found between the polymeric and exclusive enteral diets in terms of inducing remission and anti-inflammatory properties [6]. The compliance of exclusive Enteral Nutrition is low due to the fact that it is more expensive and poorer in taste.

In their study, KE Whitten et al. found that EN is not used in all pediatric centers. In the centers where EN is used, there is no consensus on the choice, quantity, way of administration, and duration of use of enteral products [24]. In many studies, modifications, which include changes in fat and protein content and type and the addition of bioactive peptides of enteral diet composition, were investigated. The TGFβ diet was influential in inducing the remission and mucosal healing. Biochemical inflammation markers, erythrocyte sedimentation rates, and C-reactive protein levels became normal, and serum albumin levels improved at a significant level. It was determined in the endoscopic examination that there were significant improvements in the appearance and histology of the mucosal tissue; and there were decreases in mRNA levels for the pro-inflammatory cytokines interleukin-1β, IL-8, and interferon-gamma. Modulen IBD, which is a nutritional supplement, was given in addition to a normal diet to provide 35%–50% of total calorie intake [25,26].

In our own clinical practice, we give Modulen IBD for eight weeks as 1000 kal/day in addition to the treatment and normal nutrition in cases with IBD.

In the studies conducted on TGF β diet administered up to eight weeks in patients with CD, TGF β was found to be effective in mucosal healing and remission induction. In addition, it was shown that CRP, sedimentation, and albumin levels were significantly improved in these patients [27,28,29]. It was reported that sedimentation values significantly improved in children receiving Modulen IBD [25]. In their study, Sigall et al. reported that sedimentation and CRP values were normalized in 70% of cases receiving EN in addition to their normal diet [30]. Similarly, Johnson et al. reported that inflammatory parameters improved in 85% of the patients receiving EN in addition to their normal diet [31]. In most children receiving EN, the energy and balance condition is improved within 7–10 days, but the continuation of the treatment is recommended. Inflammatory markers begin to fall within the first 14 days of treatment, but it is generally recommended that treatment lasts up to 6–8 weeks [32]. The mean duration of treatment in both adult and pediatric age groups was found to be eight weeks [24]. Borelli et al. found a statistically significant difference among CRP, sedimentation, and albumin levels in children with CD, who received a polymeric diet before and after treatment [33]. In their study using Modulen IBD, Buchan et al. reported that CRP significantly improved sedimentation values in the second month compared to the onset of treatment [34]. In our study, in accordance with the literature, a significant improvement was observed in platelet, serum albumin values, sedimentation, and CRP values in the group receiving Modulen IBD after the first week. The CRP values were normal in the first month, others were normalized in the third month and no statistically significant difference was found between CD-M and CD groups.

No difference was found between the elemental diet, the polymeric diets, and the induction rates [25], and it was shown that the use of TGF-β-rich nutrition products was effective in mucosal healing, induction of the remission, maintenance of remission, and reduction of PCDAI [25,27,28,29]. Without limiting the normal diet of children, it was observed that providing EN support prolonged the remission period and improved growth [35,36,37].

In one study, clinical remission was obtained eight weeks after treatment in 79% of the children receiving polymeric diet [7]. Borelli et al. found a significant difference in PCDAI before and after treatment in children with CD receiving polymeric diet [33]. In many studies, relapse rates were reported to be higher providing that the intestinal inflammation persisted after remission induction [33]. No difference was found among the remission rates of elemental, semi-elemental, and polymeric diets in meta-analysis [38]. Rubio et al. used Modulen IBD approximately for two months and reported a significant reduction in PCDAI with a remission rate of 76% for oral intake and up to 85% with nasogastric intake [39]. Buchanan et al. used Modulen IBD for eight weeks and reported remission rates up to 80% [34]. Similarly, Fell et al. also found a clinical remission rate of 79% in a study in which they investigated the efficacy of treatment with TGF β [7]. In three previous studies, children were evaluated about the role of prolonged supplementation with liquid diets to maintain remission and growth improvement. These studies argued that supplementary enteral nutrition without restricting the normal diet was associated with prolongation of the remission and improved linear growth [1,35,36].

In our study, PCDAI was higher in the CD-M group. We determined that it started to improve and completely normalized in three months. In the control group, PCDAI was in progress in three months and a statistically significant difference was observed. While no patient was in remission in the first week, one case in the first month and 14 (88%) cases in three months were in remission in CD-M group. In the control group, eight patients (42%) were able to be in remission. All patients were in remission in the sixth month. Our remission rates were in consistent with the literature.

Weight and height retardation were determined in 85% of the children with CD at the time of diagnosis; and 20%–30% was reported to have continued growth retardation when they reached adulthood [17,40]. In their study, Borelli et al. showed that the polymeric diet indicated an increase in weight and height scores before and after treatment in children with CD; however, no significant difference was found between them [33]. Similarly, in the study of Day et al., it was shown that there was a significant improvement in weight and height z scores of the patients but there was no statistically significant difference [41]. In the study of Knight et al., it was reported that the improvement was observed in weight z score whereas there was no improvement in height z scores [42]. Buchanan et al. reported that there was a significant improvement in weight and height z scores of TGF-rich polymeric diet [34]. It has been reported that weight gain is better than elemental formulas in the use of polymeric formulas [43]. In our study, weight z scores were significantly lower in the CD-M group, although no difference was found between the height z scores at the time of diagnosis in both groups. In the CD-M group, the weight z score started to improve in the first week after the EN supplementation and reached normal values in three months and there no significant difference was observed between the two groups in the third month. At the end of the first month, the height z score started to improve in the CD-M group and there was no statistically significant difference between the two groups in the third month. Summary of the studies conducted on the effect of Modulen IBD on clinical, laboratory, and anthropometric values of CD cases is showed on the Table 5.

Growth retardation was detected in 7–9% of children with UC and in 22–24% of children with CD in a study conducted with 783 IBD patients [45]. Although growth retardation was detected in both UC and CD patients, it was detected less commonly in UC patients [46,47]. Different from CD, there are few studies conducted on the efficacy of EN on remission induction, remission maintenance, inflammatory parameters, and anthropometric values in patients with UC. In a study conducted on this subject where EN and total parenteral nutrition were compared, serum albumin values were observed more frequently in EN, and no differences were detected between anthropometric values although serum albumin values were higher in EN [48]. In another study conducted on patients with UC, significant increases were detected in serum prealbumin values; however, no changes were detected in other laboratory parameters [49].

Unlike CD cases, there are no data on the use of EN alone as the primary treatment method in acute severe diseases. However, it was seen that patients’ weight loss stopped and all nutritional and clinical parameters improved when given in addition to standard treatment [44]. Our study showed that, although not as much as in CD patients, TGF-β support induced the remission of the patients in patients with UC and contributed to the early improvement of inflammatory and anthropometric parameters in these patients.

One of the limitations of the present study of ours was that the endoscopic and histopathological evaluation of the intestinal mucosa could not be performed after treatment because of the retrospective nature of our study. Therefore, the histological healing of the mucosa could not be evaluated.

One of the strengths of our study was that it is one of the first studies that evaluated in detail the effects of Modulen IBD given to patients with CD in addition to initial medical treatment and without limiting their daily diet. This is the first study that evaluated the effects of Modulen IBD given to patients with UC in addition to their initial medical treatment and without limiting their daily diet. We believe that modular IBD given without any limitations in the daily diets increased oral tolerance.

## 5. Conclusions

Our study showed that TGF-β-rich enteral nutrition support in children with IBD is an easy, effective, and reliable approach. In the light of these data, TGF-β-rich enteral nutritional support given in addition to the medical treatments without restricting normal diets avoided the potential side effects of steroids in CD patients who received steroids had remission earlier, and remained in remission for longer durations, and contributed to improve the weight and height scores earlier. Our study showed that TGF β-rich enteral nutrition solution had positive contributions in UC patients and was beneficial in reducing the severity of the disease and in the treatment and prevention of growth retardation; and it may be an alternative option in the treatment of children with malnutrition.

## Figures and Tables

**Table 1:** Laboratory parameter comparison of cases with Crohn disease (CD)-Modulen and CD group.

Patients Marker	Start of Modulen IBD (Mean ± SD)	P^1^	1 Week after Modulen IBD (Mean ± SD)	P^2^	1 Month after Modulen IBD (Mean ± SD)	P^3^	3 Months after Modulen IBD (Mean ± SD)	P^4^	6 Months after Modulen IBD (Mean ± SD)	P^5^
Leucocyte count-Modulen IBD	9302 ± 2427	0.596	10,056 ± 3251	0.703	12414 ± 5555	0.497	10,530 ± 5526	0.185	9512 ± 3693	0.280
Leucocyte count (Range: 4.5–11.5 × 10^3^/µL)	9254 ± 4729		10,230 ± 332		11,645 ± 6378		8368 ± 2843		8095 ± 3120	
Hemoglobin-Modulen IBD	10.7 ± 1.8	0.021	16.7 ± 23	0.164	11.6 ± 2	0.169	11.3 ± 2	0.010	11.9 ± 1.83	0.088
Hemoglobin (Range: 12–16 g/dL)	12.1 ± 2.3		12.2 ± 1.9		12.6 ± 2.3		13.3 ± 1.8		13 ± 2	
Platelets count-Modulen IBD	424,687 ± 12,970	0.030	415,812 ± 108,789	0.030	406,562 ± 112,316	0.018	387937 ± 128979	0.241	385312 ± 137445	0.282
Platelets (Range:150–450 × 10^3^/µL)	362,736 ± 17 9,751		343,684 ± 112,707		320,555 ± 85,996		333833 ± 122967		324,526 ± 78,080	
Total protein-Modulen IBD	6.6 ± 0.8	0.357	6.7 ± 0.7	0.443	6.7 ± 0.6	0.386	6.9 ± 0.7	0.903	6.8 ± 0.3	0.014
Total protein (Range: 5.6–6.8 g/dL)	6.8 ± 0.7		6.9 ± 0.7		6.9 ± 0.4		7 ± 0.7		7.2 ± 0.4	
Albumin-Modulen IBD	3.13 ± 0.7	0.007	3.4 ± 0.6	0.009	3.66 ± 0.45	0.018	3.8 ± 0.4	0.326	4.1 ± 0.3	0.894
Albumin (Range:3.5–5.5 g/dL)	3.8 ± 0.6		3.87 ± 0.4		4 ± 0.4		4 ± 0.4		4.2 ± 0.4	
ESR-Modulen IBD	57 ± 26	0.17	42 ± 28	0.331	19.8 ± 13	0.037	8.7 ± 5.8	0.504	6 ± 3.8	0.880
ESR (Range: 0–20 mm/h)	43.8 ± 17		33.6 ± 12.9		29.9 ± 13		8.8 ± 2.4		6.2 ± 3.6	
CRP-Modulen IBD	5.4 ± 6.1	0.38	2.3 ± 2	0.068	2.8 ± 3.9	0.172	1 ± 1.9	0.777	0.3 ± 0.2	0.250
CRP (Range: 0–2 g/L)	2.9 ± 3.4		1.4 ± 1.7		1 ± 2.1		0.6 ± 0.7		0.9 ± 1.4	

P^1^,P^2^,P^3^,P^4^ and P^5^ are the *p* values in initial Modulen IBD treatment, one week after Modulen IBD, one month after Modulen IBD, three months after Modulen IBD, six months after Modulen IBD, respectively that were determined at the time of diagnosis after the comparison of the data of the groups that received and that did not receive Modulen IBD.

**Table 2:** Activity index, remission rate, and anthropometry comparison of cases with CD-Modulen and CD group.

Patients Marker	Start of Modulen IBD	P^1^	1 Week after Modulen IBD	P^2^	1 Month after Modulen IBD	P^3^	3 Months after Modulen IBD	P^4^	6 Months after Modulen IBD	P^5^
PCDAI-Modulen IBD (mean ± SD)	54.2 ± 7	0.003	48 ± 6.8	0.006	16.7 ± 4.8	0.001	7.5 ± 2.5	0.001	7.2 ± 3.5	0.167
PCDAI (mean ± SD)	44.4 ± 10.3		40 ± 8.7		34.8 ± 7.6		23 ± 5.5		8.7 ± 3.6	
Remission induction rate-Modulen IBD (*n*/*N*; %)					1/16 (70%)	0.235	14/16 (88%)	0.001	16/16 (100%)	1
Remission induction rate (*n*/*N*; %)							8/19 (42%)		19/19 (100%)	
Height for age z score-Modulen IBD (mean ± SD)	−0.2 ± 1.2	0.822	−0.09 ± 1.4	0.862	−0.01 ± 1.5	0.781	0.08 ± 1.5	0.768	0.87 ± 0.83	0.370
Height for age z score (mean ± SD)	0.06 ± 1.3		0.08 ± 1.3		0.21 ± 1.3		0.28 ± 1.25		0.5 ± 1.2	
Weight for age z score-Modulen-IBD (mean ± SD)	−2.2 ± 1.6	0.016	−1.18 ± 1.1	0.014	−0.89 ± 1.43	0.050	0.05 ± 0.93	0.655	0.64 ± 0.78	0.596
Weight for age z score (mean ± SD)	1.28 ± 1.2		−0.06 ± 1.65		0.13 ± 1.69		0.32 ± 1.8		0.58 ± 1.79	

P^1^,P^2^,P^3^,P^4^ and P^5^ are the *p* values in initial Modulen IBD treatment, one week after Modulen IBD, one month after Modulen IBD, three months after Modulen IBD, six months after Modulen IBD, respectively that were determined at the time of diagnosis after the comparison of the data of the groups that received and that did not receive Modulen IBD.

**Table 3:** Laboratory parameter comparison of cases with ulcerative colitis (UC)-Modulen and UC group.

Patients Marker	Start of Modulen IBD (Mean ± SD)	P^1^	1 Week after Modulen IBD (Mean ± SD)	P^2^	1 Month after Modulen IBD (Mean ± SD)	P^3^	3 Months after Modulen IBD (Mean ± SD)	P^4^	6 Months after Modulen IBD (Mean ± SD)	P^5^
Leucocyte count-Modulen IBD	9581 ± 3505	0.470	9920 ± 2545	0.518	10,600 ± 4955	0.747	10,383 ± 3058	0.176	10,786 ± 5753	0.054
Leucocyte count (Range: 4.5–11.5 × 10^3^/µL)	10,704 ± 4782		9931 ± 6214		9731 ± 5004		9253 ± 4212		8208 ± 3589	
Hemoglobin-Modulen IBD	9.2 ± 2.5	0.700	9.9 ± 2.3	0.963	10.3 ± 2.6	0.569	10.6 ± 2.3	0.782	10.9 ± 2.1	0.261
Hemoglobin (Range: 12–16 g/dL)	9.6 ± 2.4		9.9 ± 2.8		10.4 ± 2.5		10.3 ± 2.7		11.4 ± 2.5	
Platelets count-Modulen IBD	525,423 ± 164,735	0.022	483,615 ± 203,403	0.389	489,461 ± 213,085	0.470	486,461 ± 216,880	0.332	463,923 ± 191,347	0.162
Platelets (Range: 150–450 × 10^3^/µL)	436,280 ± 112,322		417,072 ± 185,701		425,400 ± 136,758		402,000 ± 115,610		353,560 ± 81,718	
Total protein-Modulen IBD	6.4 ± 1.1	0.470	6.4 ± 1	0.296	6.9 ± 0.7	0.437	6.9 ± 0.7	0.731	6.8 ± 0.6	0.256
Total protein (Range: 5.6–6.8 g/dL)	6.8 ± 0.6		6.9 ± 0.6		6.7 ± 0.8		6.8 ± 0.8		7 ± 0.7	
Albumin-Modulen IBD	3.1 ± 0.9	0.017	3.4 ± 0.5	0.066	3.7 ± 0.5	0.098	3.9 ± 0.6	0.174	3.95 ± 0.4	0.180
Albumin (Range:3.5–5.5 g/dL)	3.77 ± 0.5		3.8 ± 0.6		4 ± 0.5		4.3 ± 1.1		4.1 ± 0.1	
ESR-Modulen IBD	51.7 ± 31	0.302	36 ± 28	0.020	17.9 ± 9.8	0.004	9.3 ± 4.5	0.828	8.5 ± 6.5	0.463
ESR (Range: 0–20 mm/h)	58.5 ± 25		50 ± 21		30.8 ± 11.3		11 ± 7.9		6 ± 3.3	
CRP-Modulen IBD	1.6 ± 1.8	0.248	0.88 ± 1.39	0.017	0.65 ± 1	0.026	0.66 ± 0.51	0.440	0.45 ± 0.4	0.910
CRP (Range: 0–1 g/L)	2.3 ± 2.1		2.2 ± 2.9		2.3 ± 3.3		1.89 ± 3.8		0.8 ± 0.9	

P^1^,P^2^,P^3^,P^4^ and P^5^ are the *p* values in the initial Modulen IBD treatment, one week after Modulen IBD, one month after Modulen IBD, three months after Modulen IBD, six months after Modulen IBD, respectively that were determined at the time of diagnosis after the comparison of the data of the groups that received and that did not receive Modulen IBD.

**Table 4:** Disease activity index, remission rate, and anthropometry comparison of cases with UC-Modulen and UC group.

Patients Marker	Start of Modulen IBD	P^1^	1 Week after Modulen IBD	P^2^	1 Month after Modulen IBD	P^3^	3 Months after Modulen IBD	P^4^	6 Months after Modulen IBD	P^5^
PUCAI-Modulen IBD (mean ± SD)	58.8 ± 13.8	0.698	54.9 ± 12.9	0.698	26.9 ± 5.9	0.001	15 ± 3.3	0.001	8.5 ± 5.6	0.187
PUCAI (mean ± SD)	56.4 ± 15.3		53.8 ± 13.9		46 ± 9		29 ± 13		9.6 ± 4	
Remission induction rate-Modulen IBD (*n*/*N*; %)					3/13 (21%)-	0.001	8/13 (62%)	0.001	13/13 (100%)	1
Remission induction rate (*n*/*N*; %)							4/25(16%)		25/25 (100%)	
Height for age z score-Modulen IBD (mean ± SD)	−1.23 ± 1.68	0.030	−1.29 ± 1.6	0.017	−0.9 ± 1.75	0.069	−1 ± 1.7	0.009	0.4 ± 1	0.558
Height for age z score (mean ± SD)	−0.05 ± 1.5		−0.1 ± 1.5		0.08 ± 1.5		0.24 ± 1.6		0.3 ± 1.5	
Weight for age z score-Modulen-IBD (mean ± SD)	−1.02 ± 1.9	0.029	−0.94 ± 1.8	0.056	−074 ± 1.61	0.096	−0.06 ± 0.8	0.924	0.4 ± 0.7	0.201
Weight for age z score (mean ± SD)	−0.46 ± 1		−0.5 ± 0.9		−0.3 ± 1		−0.21 ± 1		−0.03 ± 1	

P^1^,P^2^,P^3^,P^4^ and P^5^ are the *p* values in the initial Modulen IBD treatment, one week after Modulen IBD, one month after Modulen IBD, three months after Modulen IBD, six months after Modulen IBD, respectively that were determined at the time of diagnosis after the comparison of the data of the groups that received and that did not receive Modulen IBD.

**Table 5:** Summary of the studies conducted on the effect of Modulen IBD on clinical, laboratory, and anthropometric values of CD cases.

References		Disease Group	Effects of Modulen IBD
Buchan et al.	[34]	CD	Improvement in weight z score, sedimentation, decrease in CRP and clinical remission
Heuschkel et al.	[27]	CD	Improvements in growth and development, CRP, sedimentation and albumin values
Hartman et al.	[25]	CD	Decrease in sedimentation and CRP, normalization of weight and height z scores, decrease in PCDAI, continuing remission
Rubio et al.	[39]	CD	Decrease in sedimentation, CRP, albumin, hemoglobin and thrombocyte values, decrease in PCDAI, induction and continuation of remission, increase in weight and height
Fell et al.	[7]	CD	Decrease in CRP sedimentation, improvements in endoscopic and histologic mucosal terms, improvement in growth, induction and continuation in remission
Day, A.S. et al.	[32]	CD	Improvement in sedimentation, CRP and albumin values, thrombocyte values, increase in weight and height and continuation and induction in remission
Borelli et al.	[33]	CD	Improvement in sedimentation, CRP and albumin values, decrease in PCDAI, induction and continuation of remission, increase in weight gain
Triantafillidis et al.	[44]	CD	Improvement in sedimentation, CRP, albumin, ensuring clinical remission, increase in weight and height
